# Supplementary material for: Reduction in Phosphoribulokinase Amount and Re-Routing Metabolism in Chlamydomonas reinhardtii CP12 Mutants
Source: Int J Mol Sci. 2022 Feb 28;23(5):2710. doi: 10.3390/ijms23052710 (PMC8910624; doi:10.3390/ijms23052710)
Supplement: Supplementary file 1 [file ijms-23-02710-s001.zip › Supplementary-Figures.pdf]

Supplementary Material

(a)

```
ATCACTTGAGCAAACGAAATGCCTCAGAAAAACGCTGACCGTCTTCAGCGGCCTATCGAAAAAAGCGGGTCCCTCTCGCGAGCAT
TTCGCAAACGCTTAAACTCCAACCAGCTCGTCGGTCTCGCCGCAAGCGCCCAGCGAGCGGGCGCCTTACTCAACCGGCTGCCACG
CCTGCGTTTTAGCCCCTACGCCACTGGTTGCGACCCCTTATCGCTAAGACGTACGGCCGGCCCATGCAGCCCGCTGCGAGCCGGT
                                                                     M  Q  P  A  A  S  R
TGATAAAGAAGTTCCGCACCGTGCCCGGCGACAAACACACTATTACAAAAATGATGCTCACTAAGTCCGTTGTTATTAGCCGGCC
L  I  K  K  F  R  T  V  P  G  D  K  H  T  I  T  K  M  M  L  T  K  S  V  V  I  S  R  P
GGCCGTGCGCCCTGTCTCCACCCGCCGCGCGGTGGTGGTCCGCGCTAGCGGCCAGCCCGCTGTGGACCTGAACAAGAAGGTTCAG
  A  V  R  P  V  S  T  R  R  A  V  V  V  R  A  S  G  Q  P  A  V  D  L  N  K  K  V  Q
GACGCCGTGAAGGAGGCTGAGGACGCTTGCGCCAAGGGCACCTCCGCGGTGAGGCGTTACTACTAGTCGCTTCAGTGGGGGCCAC
 D  A  V  K  E  A  E  D  A  C  A  K  G  T  S  A
ATCATCGGGCTTGAAAGGATCCTGGCTCGTCGGAACGCTTGCGCACACCCAGTTGGTGTTTCTCCGCGTCCTGATCCCAGCTAGG
CTTCATTTCAGTGTGTTACGATCAAGGAGACAGTAGATCACGCGGCGCTCGGCCTGGCAGTGGTCGATATATCGCGGACCGCTCC
CTTCGCTTGCACAACGTTGCGTCTTGTTTTTCTTGACTCGAGCCTAAACACGCTCCTGTATTTTCATATACAGGACTGCGCCGTG
                                                                          D  C  A  V
GCCTGGGACACCGTTGAGGAGCTCAGCGCTGCTGTCTCGCACAAGAAGGATGCCGTCAAGGCCGACGTCACCCTGACCGACCCCC
 A  W  D  T  V  E  E  L  S  A  A  V  S  H  K  K  D  A  V  K  A  D  V  T  L  T  D  P
TGGAGGCTTTCTGCAAGGATGCCCCCGATGCCGATGAGTGCCGGTGAGTAGCCGGCCTGAATTGTCGCTCTGCGCGTGTCGGCAT
L  E  A  F  C  K  D  A  P  D  A  D  E  C  R
TCGATCGCTCAGAAGTTCTCCCACATTTCCGAGACGACCTGCCTGAACAGCACCTCCTGGCTGCATCACATGCCAGTGATAGCTG
TGAATGCGTATTCGCTGGGTTGAGGTCTCGACTCAAGGGTGTTGCACCCCGGCCACCACTAGGGTTCCGTTCGGGCGCCTGGGAC
CCAGCCAACGCGCTTGCCGCATACCAACAGCGGCGTAACCTGCTCTCAATCTGTATGGCTGTCGTGACCCGTGTGTTTTCCTCTC
CCCGGTGCCCTGCCCCAACAGCGTCTACGAGGATTAAGCAGCTCACCCAACACGCTTTGGGCGGGCGGCAAGGCCTCGTGGCAGC
                          V  Y  E  D  *
AGCGGCGCTAGTGCAGCACGTTGGTGCCTTGAGGAAGGACAACTAGGTGGTTGAGTGGCGCACGGTATGGCGGGTTGCACAATGC
AACCGGCCAGCTGGGCGAAGGGAGTTCGAGCGGTGCAGGTGTGTGGGAGCCTGGAGCCCGCGTTCCGTCGGACGTTCGCCGCGTG
CAGATTTGCGCAGCTGGCGCTTCTTTTCGGAGTTTTCGGGCTAGGATAGGACCAGCCCTTGATCGTGGCAGACCCGAGCAGTTCG
GAGCGATGTGGTGAGCAGCAGGGCCGGGCCGGGCGTCCGGCTCCGTGTCCTGTAGTTTTGGTGTGGGGCACTGTTTGGCCCCGCT
TGTACCGGTTTCCCCTGTTGTACAGAGTCGTCGGTGTCGACACATGATGAAAGGAATTCATGCATCATGGTACAAAATAATGAAT
GTAACGGCGCAAGCCGTGCACGCACAAGCTGTCCTGATGATAGCGTAATGGTTTCAATGCGGGCTGCCGTTCATGAGGATGGGTT
CAGTGACAATCTGGGTGGGCAGCGCATGGAGCGTGAAGAAATAGCAGTGACCAGCCAGCAGGAAAACTCCTGCCTACGGGAATCT
CAGCCAAGGAATGCGGCATGGCGAAGCCTGTCAGCAACCCGACACGGCGAAGCAGTGACCACGGCCAAGCTACAGCGGCTCAGTA
GCACGATTAGCTTTTGCGCCAGGCGCACGTGAGCGCACTGCTTTGTATTCACGCCTGCTATCTGTTTCCACACTTGCCTTCGCCC
ACGCCTTCACCTGCAACTTCCGTGTTCGTCAAC
```

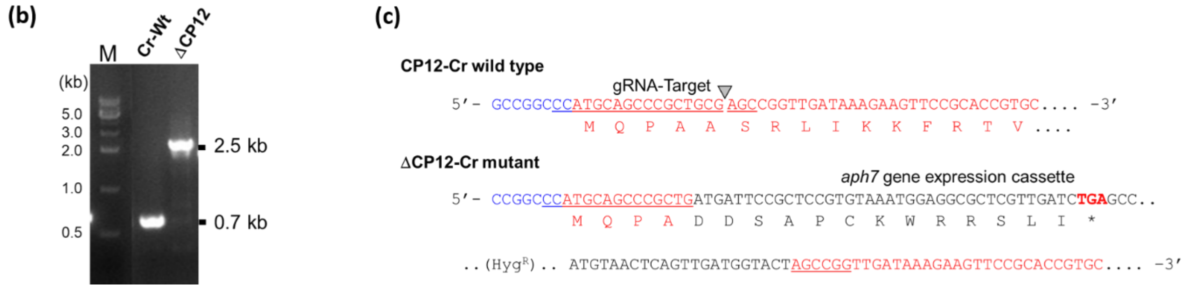

Figure S1: The analysis of the genome sequence ΔCP12-Cr and WT-Cr (**a**) The genomic sequence (Cre08.g380250) of WT-Cr. Blue and black letters indicate the untranslated region and intron sequence, respectively, and red letters represent exon and amino acid sequences. The underline indicates the twenty bp-long gRNA target sequence. (**b**) To confirm targeted mutation of the CP12 gene, cells were subjected to genomic PCR with specific primers adjacent to sgRNA target sites in wild type and ΔCP12-Cr. (**c**) The edited genome sequence of a ΔCP12-Cr . The genotype of CP12 was determined by Sanger sequencing. The ΔCP12-Cr has a DNA fragment of the *aph7* gene expression cassette inserted within the gRNA target sequence.

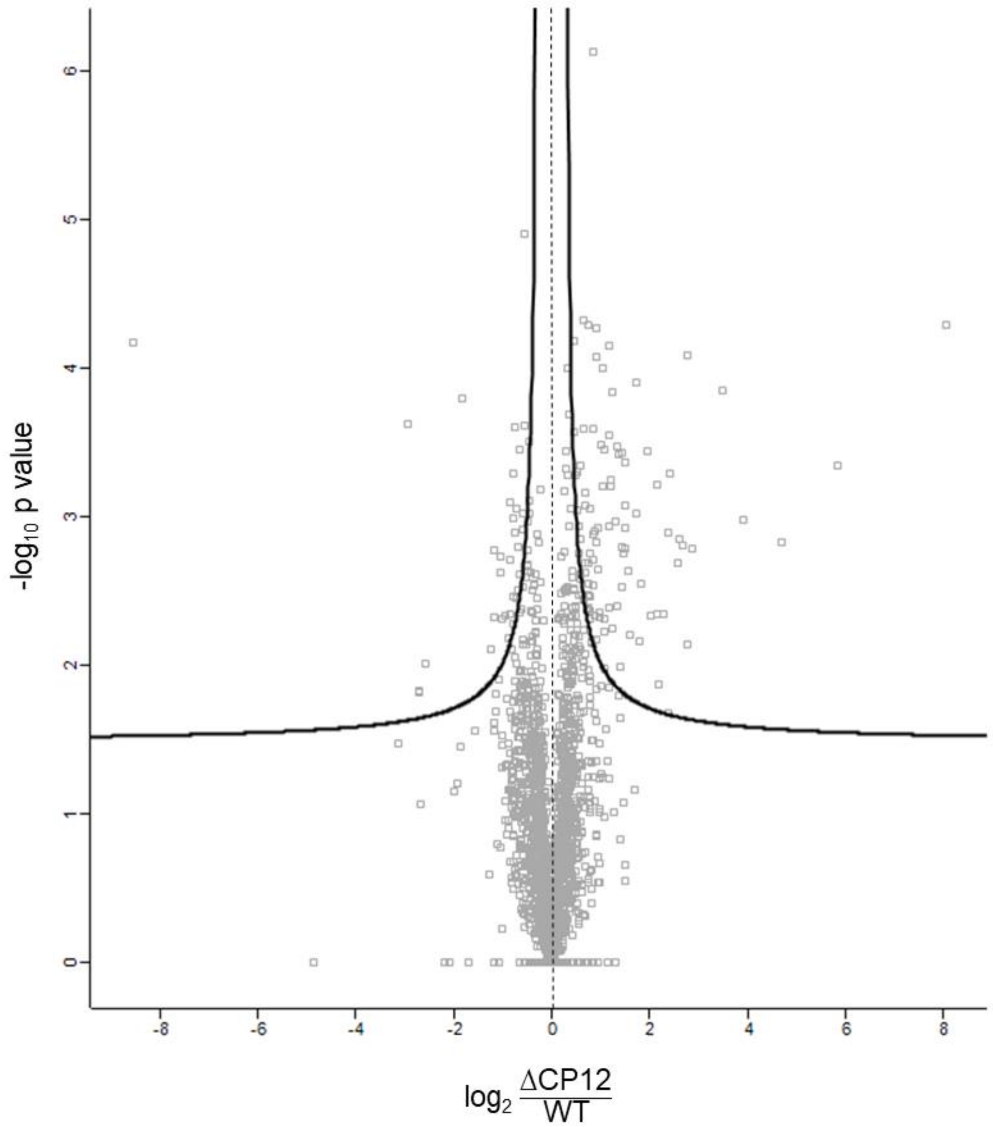

Figure S2: Volcano plot of the proteomic data. The plot represents the fold changes in relative abundance for each protein (empty square). The x-axis shows difference of protein abundance in ΔCP12-Cr versus WT-Cr (in log_2). The y-axis shows the $-log_{10}$ *p* value. The dotted vertical line indicates unchanged protein amount. The continuous lines show the threshold above which the proteins are in statistically significant differential amount (two-sample *t*-test with a permutation-based false discovery rate set at 0.01 and *p*-value adjusted using a scaling factor s0 to a value of 0.1) that are on the two top left and top right corners.

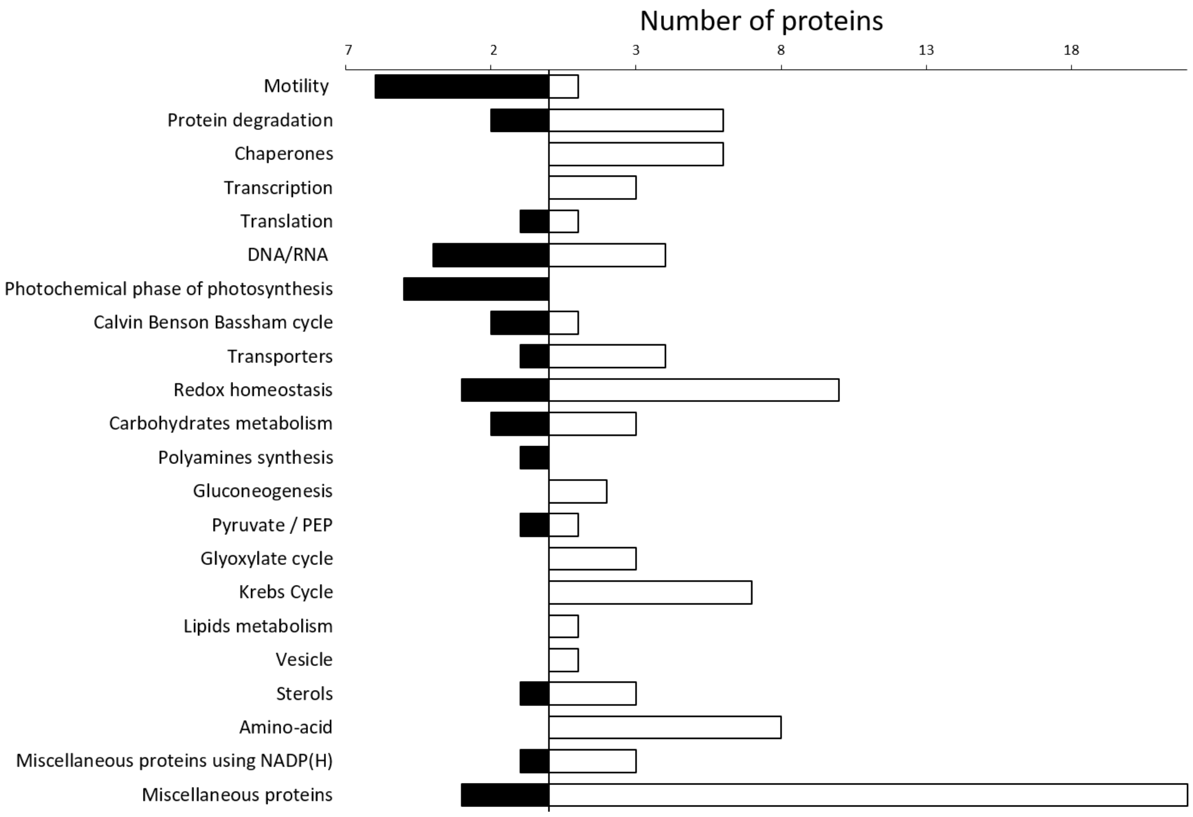

Figure S3: Number of proteins increasing or decreasing in ΔCP12-Cr strain, all metabolic pathways are shown. The proteins that are more abundant in ΔCP12-Cr compared to WT-Cr are counted in the white bars, the protein that are less abundant in ΔCP12-Cr compared to WT-Cr are counted in the dark bars.

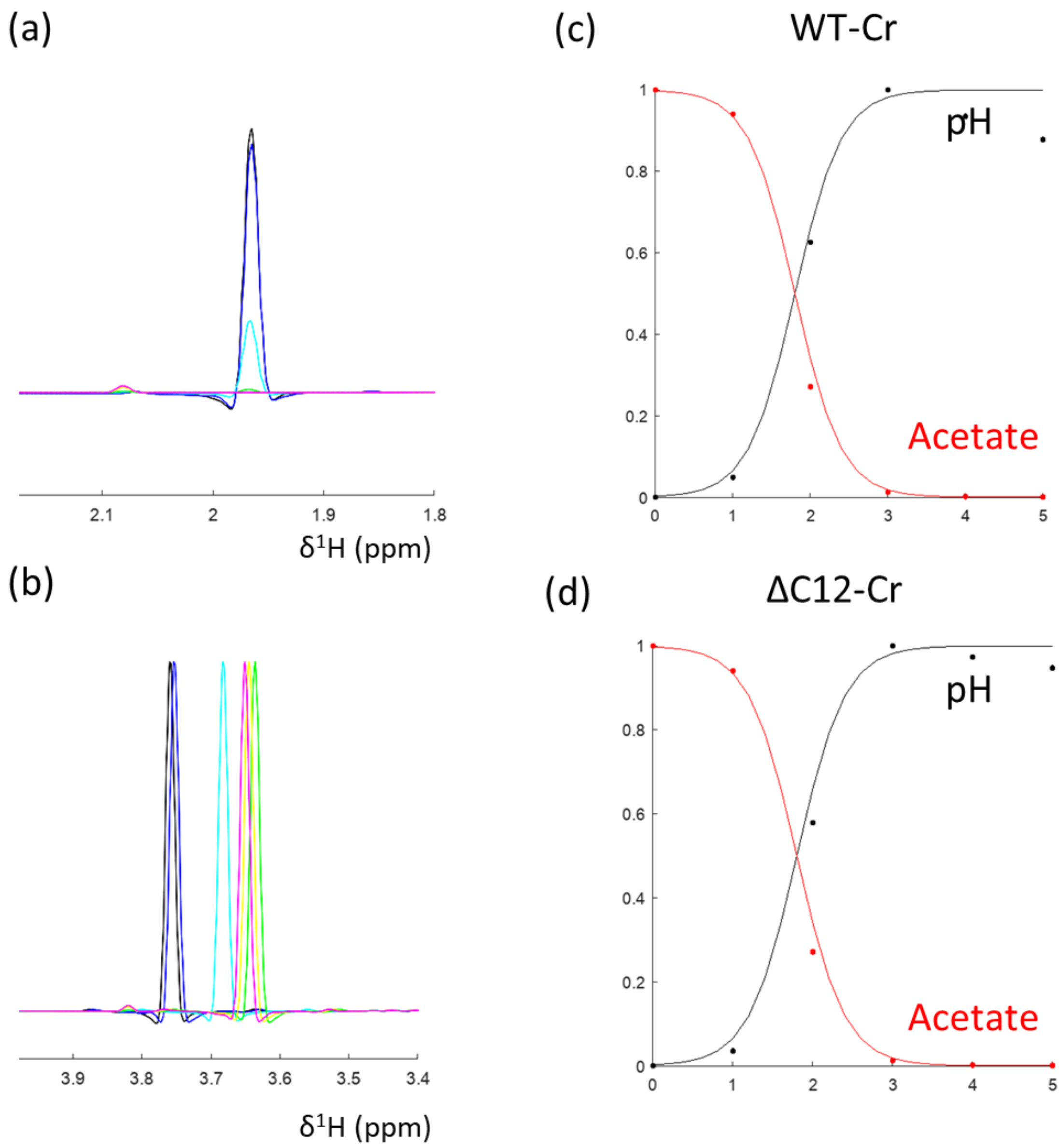

Figure S4: Acetate concentration and pH variation in mixotrophic precultures. (**a** and **b**) Superimposition of ^{1}H 1D NMR spectra of culture medium collected at time 0 (black), and after 1, 2, 3, 4 and 5 days of pre-culture (in blue, cyan, green, yellow and magenta respectively). In (**a**), the region of the acetate methyl resonance is shown. The decrease in intensity of this resonance is related to the consumption of actetate during mixotrophic growth. In (**b**), the region of the Tris(hydroxymethyl)aminomethane methylene resonance is shown. The shift of this resonance is related to a variation of pH from 7.4 to a more basic value because of photosynthesis CO_2 consumption during mixotrophic growth. (**c** and **d**) Relative acetate amount and pH variation during mixtrophic growth of WT-Cr (**c**) and ΔCP12-Cr (**d**). After four days of pre-culture, the acetate has been consumed from the medium. We collected these cultures after five days, when no acetate is left in the medium, and then subcultured them in phototrophic condtions.
